# Personal Correlates of Support for Medical and Recreational Cannabis Legalization in Australia

**DOI:** 10.3389/fpsyt.2021.551661

**Published:** 2021-02-25

**Authors:** Vivian Chiu, Gary Chan, Wayne Hall, Leanne Hides, Carmen Lim, Janni Leung

**Affiliations:** ^1^School of Psychology, University of Queensland, Brisbane, QLD, Australia; ^2^National Centre for Youth Substance Use Research, University of Queensland, Brisbane, QLD, Australia; ^3^National Drug and Alcohol Research Centre, University of New South Wales Sydney, Sydney, NSW, Australia

**Keywords:** cannabis (marijuana), opinion, attitudes, determinants, marijuana

## Abstract

**Introduction and Aims:** Increasingly more Australians are in favor of legalizing medical and recreational cannabis use. This paper explored the personal characteristics of those who supported each of these policies in Australia.

**Design:** Cross-sectional national survey.

**Methods:** This study included 21,729 participants aged 18 years and above who responded to the 2016 National Drug Strategy Household Survey. Participants were provided the assurance of confidentiality for their participations. Logistic regression models were used to examine the relationships between personal characteristics and support for the legalization of medical and recreational cannabis.

**Results:** Overall, 77 and 40% of participants supported the legalization of medical and recreational cannabis respectively. People of older age were more likely to support medical cannabis legalization while those who supported legalization of recreational cannabis use were more likely to be younger. Medical cannabis supporters were more likely to report chronic pain (OR = 1.44, 95% CI: 1.04, 2.00) while recreational cannabis supporters were more likely to suffer high level of psychological distress (OR = 1.28, 95% CI: 1.14, 1.43). Experience with cannabis use was strongly associated with supportive attitudes, with recent cannabis users almost 14 times (OR = 14.13, 95% CI: 5.37, 37.20) and 34 times (OR = 33.74, 95% CI: 24.22, 47.01) more likely to support the legalization of medical and recreational cannabis use, respectively.

**Discussion and Conclusions:** The majority of Australians approve the legalization of cannabis for medicinal purposes but most remain cautious about legalizing recreational cannabis use. The sociodemographic and clinical profile of supporters of medical and recreational legalization suggests a potential interaction of self-interests and beliefs about the harms of cannabis use.

## Introduction

Australian support for the legalization of medical cannabis has been stable for a decade since the 2000s with 68.5–69% of persons supporting legalization between 2004 and 2013 (1, 2) despite substantial international policy changes over the period. The 2016 National Drug Strategy Household Survey (NDSHS) found an increase in support for legalizing the medicinal use of cannabis (3). This shift in attitude coincided with the Australian Federal and state governments legalized access to medicinal cannabis in the same year. So far, the growth in public support for legalization of medical cannabis use has not been accompanied by an increase in support for the legalization of recreational cannabis use, something that most Australians continue to oppose (3).

News media coverage of cannabis issues is potentially a factor that may have contributed to these shifts in public attitudes (4–6). The increased reporting of positive media stories on medical uses of cannabis may have portrayed cannabis in a more favorable light, differentiating “medical” from “recreational” cannabis despite the fact that some cannabis products are used for both purposes. The perceived health benefits of cannabis use have been highlighted by a number of studies, reporting that medical cannabis is a valid treatment for chronic pain, cancers and mood disorders (7–11). Beliefs about the medical benefits of cannabis seem more salient for supportive attitudes toward medical cannabis legalization than beliefs about its negative side effects (4). Self-medicating cannabis users are more likely to have positive views about cannabis and to describe cannabis as being less harmful than never-users (11). Individuals who have used cannabis also hold a more permissive view toward cannabis legalization (12). The official approval of medical cannabis use may be perceived as a validation of its medical value and may reduce the perceived harmfulness of cannabis use. In the United States, young adults from states that have implemented medical cannabis laws are more likely to believe that cannabis has no or low health risks than residents of states without medical cannabis laws. However, the passage of medical cannabis laws does not appear to have affected the perceived wrongfulness of recreational cannabis use (13).

There is limited information on the characteristics of Australians who support different cannabis policies. Our study contributes to the literature by analyzing correlates of support for different cannabis policies in a representative sample of the Australian general adult population. The present study used data from the 2016 National Drug Strategy Household Survey (NDSHS) to characterize the supporters of medical and recreational cannabis legalization.

## Methods

### Data Source

The study utilized data from the latest NDSHS. These data were collected between 18 June and 29 November 2016, from all Australian states and territories. The cross-sectional population survey aimed to provide reliable estimates of public awareness, attitude, and behaviors related to alcohol, tobacco, and illicit drug use in Australians 14 years and older.

### Sample Design

The NDSHS sample was selected using stratified, multistage random sampling. The sample was stratified by region (15 strata in total–capital city and rest of state for each state and territory, with the exception of the Australian Capital Territory, which operated as one stratum). To produce reliable estimates for the smaller states and territories, sample sizes were boosted in Tasmania, the Australian Capital Territory and the Northern Territory. Weighting was applied to adjust for imbalances arising from execution of the sampling and differential response rates, and thereby ensure that the results were representative of the Australian population.

### Study Population

A total of 23,772 participants completed the survey (response rate 51.1%). Of these, 18,528 (77.9%) completed the survey on paper, 5,170 (21.8%) online and 74 (0.3%) via telephone interview. This study included 21,729 participants aged 18 years and above, who responded to the questions about their support for medical and recreational cannabis legalization (91.4% of the full sample).

### Attitudes Toward Medical Cannabis Legalization

The items assessing attitudes toward medical and recreational cannabis legalization were taken from the NDSHS questions “Thinking now about the use of marijuana/cannabis for medical purposes, to what extent would you support or oppose measures such as a change in legislation permitting the use of marijuana for medical purposes?” and “Considering marijuana/cannabis, to what extent would you support or oppose the personal use of marijuana/cannabis being made legal?”, respectively. The six-point Likert scale responses was collapsed into three levels: “support” (derived from “strongly support” and “support”), “neutral” (derived from “neither support nor oppose” and “don't know enough to say”) or “oppose” (derived from “strongly oppose” and “oppose”).

### Personal Characteristics

Personal characteristics variables were chosen based on a review of studies of public attitudes toward cannabis use (7, 8, 11).

Sociodemographic characteristics included: age (age groups: “18–29 years old,” “30–49 years old” or “50+ years old”), sex (“male” or “female”), marital status (“never married,” “divorced, separated, or widowed” or “married”), education attainment (“below high school,” “high school or post-high school” or “tertiary education”), employment status (“currently employed,” “unemployed” or “not in labor force or looking for work”) and personal income [weekly income matched with national census in 2016 (Australian Bureau of Statistics): “1st quartile: nil or negative income-$399,” “2nd quartile: $400–799,” “3rd quartile: $800–1,499” or “4th quartile: $1,500 and above” per week].

Clinical characteristics included a self-reported diagnosis or treatment for cancer (“no” or “yes”) and chronic pain (“no” or “yes”) in the past 12 months. Psychological distress in the past month was assessed with the 10-item Kessler Psychological Distress Scale (K10) (14). The total score was used to define “low” (K10 score <15), “moderate” (K10 score between 15 and 20) or “high or very high” levels of distress (K10 score >21).

Cannabis use status was classified into “never user” (those who never used cannabis), “past user” (those who used cannabis but not in the past 12 months) or “recent user” (those who used cannabis in the past 12 months). Alcohol use status was defined using the Alcohol Use Disorder Identification Test (AUDIT-Consumption). The AUDIT-C is a three-item alcohol screen that consists of a scoring system to estimate alcohol consumption in a standard manner. The total scores from these questions categorized the risk levels of hazardous drinking and alcohol use disorders. The questions and responses in NDSHS were structured slightly differently from the AUDIT-C questions. Using an approximation, a similar scoring system was created to classify alcohol use status for our participants: “non-drinker or low-risk drinker” (total score ≤3.99 for male and ≤2.99 for female) or “high-risk drinker” (total score ≥4 for male and ≥3 for female). Questions in the AUDIT-C and NDSHS, and the scoring system are documented in Appendix A. Smoking status was derived from several items that measured frequency and quantity of smoking: “non-smoker” (those who used <100 cigarettes in a lifetime), “ex-smoker” (those who used 100 or more cigarettes in a lifetime but not in the past 12 months) or “current smoker” (those who used cigarettes in the past 12 months).

### Analysis

Cross-tabulations were used to compare the distributions of support for medical and recreational cannabis legalization by socio-demographics and health status. Design adjusted Rao-Scott Chi-Square tests were used to test the statistical significance of these sets of independent variables. Due to the large amount of missing responses for some independent variables, multiple imputation (30 iterations) was used to handle variables with missing values. Multiple imputation is an iterative form of stochastic imputation that leads to more accurate sets of estimates (15). It is considered as crucial in analysis of survey data with many non-monotone missing categorical variables. We included all independent variables as auxiliary variables (variables that may be correlated to the missing variable) in the imputation model.

The association between participants' characteristics and support for medical and recreational cannabis legalization were examined using multinomial logistic regression analyses. All analyses were conducted using SAS version 9.4 and were adjusted for weights and strata for differential selection, to match the survey samples to population sociodemographic distributions. In the weighted sample of 21,729 participants, the average age was 51 years (median = 51, age range between 18 and 84) with more females (54.7%) than males (45.3%). A full description of the study population is presented in Appendix B.

### Ethics

The access of the 2016 NDSHS data has been approved by the Australian Data Archive on behalf of the Australian Institute of Health and Welfare. This study has been exempted from ethics review under the National Statement on Ethical Conduct in Human Research and The University of Queensland policy (#2019001159).

## Results

**Figure 1:**
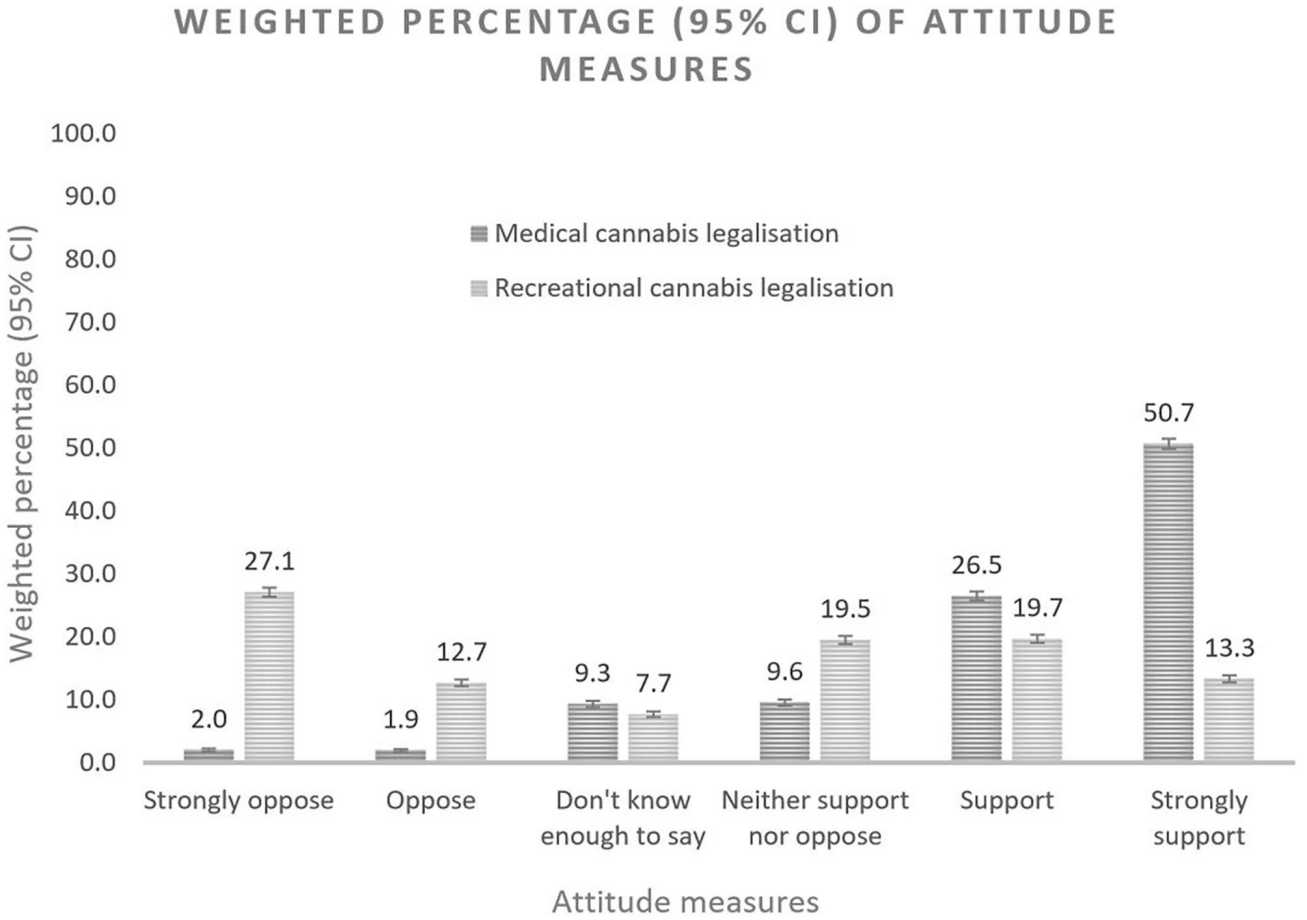
The weighted percentage (95% confidence intervals) of attitude measures.

Overall, 77% of survey participants supported the legalization of medical cannabis in 2016. In contrast, 19% of the participants were neutral and only 4% were opposed (Figure 1; Table 1). People of older age (50+ years old: OR = 1.78, 95% CI: 1.25, 2.54) and females (OR = 1.61, 95% CI: 1.33, 1.96) were more likely to support medical cannabis legalization. The association between other sociodemographic characteristics and supportive attitudes were not significant. Any personal experience with cannabis use was strongly associated with support for medical cannabis, with past users and recent users almost three times (OR = 2.78, 95% CI: 2.07, 3.73) and fourteen times (OR = 14.13, 95% CI: 5.37, 37.20) more likely to support medical use, respectively. High-risk drinking (OR = 2.12, 95% CI: 1.70, 2.65) was also associated with supportive attitudes but less so than cannabis use. Compared with participants of other health issues, people who reported having chronic pain (OR = 1.44, 95% CI: 1.04, 2.00) were more favorable to medical cannabis legalization (**Table 3**).

**Table 1:** Distribution of opinions on medical cannabis legalization distinguished by individual characteristics.

**Characteristics**		**Total** **(*N* = 21,582)**	**Support** **(*N* = 17,042)**	**Neutral** **(*N* = 3,727)**	**Oppose** **(*N* = 813)**	**χ^2^**	**df**	***p*-value**
		**Weighted %** **(95% CI)**	**Weighted %** **(95% CI)**	**Weighted %** **(95% CI)**	**Weighted %** **(95% CI)**			
Sex	Males	49.3 (48.5, 50.1)	76.0 (75.0, 77.1)	19.5 (18.5, 20.5)	4.4 (3.9, 5.0)	13.9	2	0.001
	Females	50.7 (49.9, 51.5)	78.3 (77.4, 79.2)	18.3 (17.4, 19.1)	3.4 (3.0, 3.9)			
Age group	18–29 years old	21.7 (20.9, 22.5)	71.8 (69.9, 73.8)	23.7 (21.8, 25.6)	4.5 (3.6, 5.4)	68.8	4	<0.001
	30–49 years old	35.5 (34.7, 36.3)	77.3 (76.1, 78.4)	18.7 (17.6, 19.8)	4.0 (3.5, 4.6)			
	50+ years old	42.8 (42.0, 43.6)	79.8 (78.9, 80.7)	16.6 (15.8, 17.4)	3.6 (3.2, 4.0)			
Marital status	Never married	24.2 (23.4, 24.9)	75.3 (73.6, 77.0)	20.7 (19.1, 22.3)	4.0 (3.2, 4.8)	15.4	4	0.004
	Divorced/widowed/separated	12.1 (11.7, 12.6)	80.0 (78.4, 81.6)	16.6 (15.2, 18.1)	3.4 (2.6, 4.2)			
	Married	63.7 (62.9, 64.6)	77.4 (76.6, 78.3)	18.6 (17.8, 19.4)	4.0 (3.6, 4.4)			
Employment status	Not in labor force	36.2 (35.5, 37.0)	76.4 (75.2, 77.5)	19.9 (18.8, 21.0)	3.8 (3.3, 4.3)	37.0	4	<0.001
	Unemployed/looking for work	5.9 (5.5, 6.3)	70.5 (67.1, 74.0)	24.9 (21.6, 28.2)	4.6 (3.1, 6.0)			
	Currently employed	57.9 (57.1, 58.7)	78.9 (78.0, 79.8)	17.2 (16.3, 18.1)	3.9 (3.4, 4.3)			
Education attainment	Below high school	10.8 (10.2, 11.4)	81.1 (78.8, 83.5)	15.7 (13.5, 17.8)	3.2 (2.1, 4.3)	9.5	4	0.049
	High school/post-high school	43.9 (43.0, 44.9)	80.4 (79.1, 81.6)	16.1 (15.0, 17.3)	3.5 (3.0, 4.1)			
	Tertiary	45.2 (44.3, 46.2)	78.0 (76.8, 79.3)	18.3 (17.1, 19.5)	3.7 (3.1, 4.2)			
Personal income	Lowest quartile	27.0 (26.2, 27.8)	76.4 (74.9, 78.0)	18.8 (17.4, 20.2)	4.8 (4.0, 5.6)	39.0	6	<0.001
	Medium-lowest quartile	21.6 (20.8, 22.3)	80.5 (78.9, 82.1)	16.2 (14.7, 17.7)	3.3 (2.6, 4.0)			
	Medium-highest quartile	26.6 (25.8, 27.4)	80.1 (78.6, 81.6)	16.2 (14.9, 17.6)	3.7 (3.0, 4.4)			
	Highest quartile	24.8 (24.0, 25.6)	82.8 (81.4, 84.2)	13.8 (12.5, 15.0)	3.4 (2.7, 4.1)			
Cannabis use status	Never user	63.3 (62.5, 64.1)	69.2 (68.2, 70.1)	25.6 (24.6, 26.5)	5.3 (4.8, 5.7)	725.3	4	<0.001
	Past user	26.2 (25.5, 26.9)	89.1 (88.1, 90.1)	8.8 (8.0, 9.7)	2.1 (1.6, 2.6)			
	Recent user	10.6 (10.0, 11.1)	96.4 (95.3, 97.6)	3.1 (2.1, 4.1)	0.4 (0.0, 0.9)			
Alcohol use status	Non-drinker/Low-risk drinker	55.2 (54.3, 56.0)	70.9 (69.8, 71.9)	23.8 (22.8, 24.8)	5.4 (4.8, 5.9)	392.8	2	<0.001
	High-risk drinker	44.9 (44.0, 45.7)	85.0 (84.1, 85.9)	12.8 (12.0, 13.7)	2.2 (1.8, 2.5)			
Tobacco use status	Current smoker	15.4 (14.8, 16.0)	86.0 (84.5, 87.5)	11.6 (10.1, 13.0)	2.4 (1.8, 3.1)	335.7	4	<0.001
	Ex-smoker	24.6 (23.9, 25.2)	84.6 (83.5, 85.8)	12.7 (11.6, 13.7)	2.7 (2.2, 3.2)			
	Never smoker	60.1 (59.3, 60.9)	71.9 (70.9, 72.8)	23.3 (22.4, 24.2)	4.8 (4.4, 5.3)			
Psychological distress^$^	Low level	67.8 (67.0, 68.6)	75.7 (74.8, 76.6)	20.2 (19.4, 21.0)	4.1 (3.7, 4.5)	38.3	4	<0.001
	Moderate level	20.7 (20.0, 21.3)	79.5 (78.0, 81.0)	16.6 (15.2, 18.0)	3.9 (3.2, 4.6)			
	High or very high level	11.6 (11.0, 12.1)	81.8 (79.8, 83.7)	15.0 (13.2, 16.9)	3.2 (2.3, 4.1)			
Cancer^%^	Yes	3.8 (3.5, 4.1)	81.9 (79.0, 84.9)	14.5 (11.8, 17.2)	3.6 (2.2, 4.9)	7.8	2	0.020
	No	96.3 (96.0, 96.5)	77.4 (76.6, 78.2)	18.7 (18.0, 19.5)	3.8 (3.5, 4.2)			
Chronic pain^§^	Yes	10.7 (10.2, 11.2)	85.2 (83.6, 86.9)	12.2 (10.6, 13.7)	2.6 (1.9, 3.3)	68.7	2	<0.001
	No	89.3 (88.8, 89.8)	76.6 (75.8, 77.4)	19.4 (18.7, 20.2)	4.0 (3.6, 4.3)			

*All figures are rounded to one decimal place. P-values are rounded to three decimal places*.

$ *Personal experience of psychological distress in the past month, categorized by Kessler Psychological Distress Scale (K10)*.

% *Being diagnosed or treated for cancer in the past 12 months*.

§ *Self-reported chronic pain in the past 12 months*.

Opinions about legalizing recreational cannabis were more varied, with 40% percent of Australians opposed to the policy, 33% supporting it and 27% neutral (Figure 1; Table 2). The sociodemographic profiles of persons who supported the legalization of recreational cannabis use differed from those who supported medical cannabis use. They were more likely to be younger and never married. Male and female were basically alike in their support for recreational cannabis legalization. Personal experience with substances was associated with more support for legalization of recreational cannabis use, with recent cannabis use (OR = 33.74, 95% CI: 24.22, 47.01) more strongly associated than all characteristics combined. In contrast, support for recreational cannabis legalization was significantly reduced among past cannabis users who had not used cannabis in the past 12 months (OR = 4.16, 95% CI: 3.75, 4.63). High-risk drinking (OR = 1.57, 95% CI: 1.43, 1.72) and current use of tobacco (OR = 1.47, 95% CI: 1.27, 1.70) were moderately associated with supportive attitudes. Those reporting moderate (OR = 1.59, 95% CI: 1.36, 1.85) or higher level of stress (OR = 1.28, 95% CI: 1.14, 1.43) were more supportive of legalizing recreational cannabis than those reporting low levels of stress. The results, however, suggested no association with other health conditions (Table 3).

**Table 2:** Distribution of opinions on recreational cannabis legalization distinguished by individual characteristics.

**Characteristics**		**Total** **(*N* = 20,607)**	**Support** **(*N* = 7,262)**	**Neutral** **(*N* = 6,204)**	**Oppose** **(*N* = 9,233)**	**χ^2^**	**df**	***p*-value**
		**Weighted %** **(95% CI)**	**Weighted %** **(95% CI)**	**Weighted %** **(95% CI)**	**Weighted %** **(95% CI)**			
Sex	Males	49.3 (48.5, 50.1)	35.6 (34.4, 36.8)	26.4 (25.3, 27.5)	38.0 (36.8, 39.2)	41.7	2	<0.001
	Females	50.7 (49.9, 51.5)	30.5 (29.5, 31.5)	28.0 (27.0, 28.9)	41.5 (40.4, 42.6)			
Age group	18–29 years old	21.7 (21.0, 22.5)	41.5 (39.4, 43.6)	27.9 (25.9, 29.9)	30.6 (28.6, 32.5)	339.2	4	<0.001
	30–49 years old	35.5 (34.7, 36.3)	36.9 (35.7, 38.2)	27.1 (25.9, 28.3)	35.9 (34.6, 37.2)			
	50+ years old	42.8 (42.0, 43.5)	25.5 (24.5, 26.4)	26.9 (25.9, 27.9)	47.7 (46.6, 48.7)			
Marital status	Never married	24.1 (23.4, 24.9)	44.9 (43.0, 46.8)	27.0 (25.2, 28.8)	28.1 (26.3, 29.8)	361.4	4	<0.001
	Divorced/widowed/separated	12.1 (11.6, 12.5)	30.2 (28.5, 31.9)	29.1 (27.4, 30.8)	40.7 (38.8, 42.6)			
	Married	63.8 (63.0, 64.6)	29.1 (28.2, 30.0)	26.9 (26.0, 27.8)	44.0 (43.0, 45.0)			
Employment status	Not in labor force	36.2 (35.5, 37.0)	27.1 (25.9, 28.2)	27.8 (26.6, 29.0)	45.1 (43.8, 46.4)	133.0	4	<0.001
	Unemployed/looking for work	5.9 (5.5, 6.4)	35.8 (32.1, 39.4)	29.2 (25.5, 33.0)	35.0 (31.3, 38.7)			
	Currently employed	57.9 (57.0, 58.7)	36.7 (35.7, 37.8)	26.4 (25.4, 27.3)	36.9 (35.9, 38.0)			
Education attainment	Below high school	10.8 (10.2, 11.5)	32.9 (29.9, 35.8)	28.0 (25.3, 30.6)	39.2 (36.3, 42.1)	11.4	4	0.022
	High school/post-high school	44.0 (43.0, 44.9)	35.1 (33.7, 36.6)	27.2 (25.8, 28.5)	37.7 (36.2, 39.1)			
	Tertiary	45.2 (44.2, 46.2)	35.5 (34.1, 36.9)	24.6 (23.3, 25.9)	39.9 (38.4, 41.4)			
Personal income	Lowest quartile	27.0 (26.2, 27.8)	31.9 (30.2, 33.6)	25.7 (24.1, 27.3)	42.4 (40.7, 44.2)	49.0	6	<0.001
	Medium-lowest quartile	21.5 (20.8, 22.2)	34.9 (33.0, 36.7)	27.0 (25.3, 28.8)	38.1 (36.3, 39.9)			
	Medium-highest quartile	26.7 (25.9, 27.5)	36.3 (34.6, 38.0)	27.1 (25.5, 28.6)	36.6 (34.8, 38.3)			
	Highest quartile	24.8 (24.1, 25.6)	39.2 (37.4, 40.9)	23.7 (22.2, 25.2)	37.2 (35.4, 38.9)			
Cannabis use status	Never user	63.3 (62.5, 64.1)	18.8 (18.0, 19.7)	29.2 (28.3, 30.2)	51.9 (50.9, 53.0)	2763.6	4	<0.001
	Past user	26.2 (25.5, 26.9)	46.2 (44.6, 47.7)	29.0 (27.6, 30.4)	24.8 (23.5, 26.1)			
	Recent user	10.5 (10.0, 11.0)	85.5 (83.5, 87.5)	10.5 (8.8, 12.2)	4.0 (2.8, 5.2)			
Alcohol use status	Non-drinker/low-risk drinker	55.1 (54.3, 55.9)	23.9 (22.9, 24.8)	28.0 (27.0, 29.0)	48.1 (47.0, 49.2)	771.0	2	<0.001
	High-risk drinker	44.9 (44.1, 45.7)	44.3 (43.1, 45.5)	26.3 (25.2, 27.4)	29.4 (28.3, 30.5)			
Tobacco use status	Current smoker	15.3 (14.7, 15.9)	52.0 (49.9, 54.2)	25.8 (23.9, 27.7)	22.1 (20.5, 23.8)	646.7	4	<0.001
	Ex-smoker	24.6 (23.9, 25.2)	36.6 (35.1, 38.0)	26.9 (25.6, 28.3)	36.5 (35.0, 37.9)			
	Never smoker	60.1 (59.3, 60.9)	26.7 (25.8, 27.7)	27.6 (26.7, 28.6)	45.6 (44.6, 46.7)			
Psychological distress^$^	Low level	67.8 (67.0, 68.6)	28.9 (28.0, 29.7)	27.8 (26.9, 28.7)	43.4 (42.4, 44.4)	289.0	4	<0.001
	Moderate level	20.7 (20.0, 21.4)	38.5 (36.7, 40.2)	27.0 (25.4, 28.6)	34.5 (32.8, 36.3)			
	High or very high level	11.5 (11.0, 12.1)	47.6 (45.1, 50.1)	24.5 (22.3, 26.6)	27.9 (25.7, 30.2)			
Cancer^%^	Yes	3.8 (3.5, 4.1)	27.5 (23.8, 31.1)	26.9 (23.3, 30.5)	45.6 (41.7, 49.6)	13.3	2	0.001
	No	96.2 (95.9, 96.5)	33.9 (33.0, 34.8)	27.1 (26.3, 28.0)	39.0 (38.1, 39.9)			
Chronic pain^§^	Yes	10.7 (10.2, 11.2)	36.5 (34.2, 38.8)	25.0 (22.9, 27.0)	38.6 (36.2, 40.9)	8.4	2	0.015
	No	89.3 (88.8, 89.8)	33.1 (32.3, 34.0)	27.4 (26.6, 28.2)	39.5 (38.6, 40.4)			

*All figures are rounded to one decimal place. P-values are rounded to three decimal places*.

$ *Personal experience of psychological distress in the past month, categorized by Kessler Psychological Distress Scale (K10)*.

% *Being diagnosed or treated for cancer in the past 12 months*.

§ *Self-reported chronic pain in the past 12 months*.

**Table 3:** Results of multinomial logistic regression analysis on opinions on medical and recreational cannabis legalization, using response “oppose” as reference.

**Characteristics**		**Medical cannabis legalization**		**Recreational cannabis legalization**	
		**Neutral**	**Support**	**Neutral**	**Support**
	**OR (95% CI)**	**OR (95% CI)**	**OR (95% CI)**	**OR (95% CI)**
Sex	Male	1 (reference)		1 (reference)	
	Female	1.22 (0.99, 1.50)	1.61 (1.33, 1.96)^**^	1.02 (0.93, 1.11)	0.98 (0.89, 1.08)
Age group	18–29 years old	1 (reference)		1 (reference)	
	30–49 years old	0.92 (0.65, 1.32)	1.16 (0.82, 1.63)	0.90 (0.77, 1.05)	0.94 (0.79, 1.10)
	50+ years old	0.84 (0.58, 1.22)	1.78 (1.25, 2.54)^*^	0.76 (0.64, 0.89)^*^	0.84 (0.71, 1.00)^*^
Marital status	Never married	1 (reference)		1 (reference)	
	Divorced/widowed/separated	1.03 (0.67, 1.58)	0.90 (0.60, 1.36)	0.92 (0.78, 1.10)	0.70 (0.58, 0.85)^**^
	Married	1.00 (0.71, 1.40)	0.90 (0.65, 1.24)	0.75 (0.66, 0.87)^**^	0.58 (0.50, 0.67)^**^
Employment status	Unemployed/looking for work	1 (reference)		1 (reference)	
	Not in labor force	0.74 (0.48, 1.14)	1.04 (0.69, 1.58)	0.88 (0.70, 1.10)	1.09 (0.85, 1.39)
	Currently employed	0.96 (0.63, 1.45)	1.21 (0.81, 1.81)	0.91 (0.73, 1.14)	1.08 (0.85, 1.37)
Education attainment	Below high school	1 (reference)		1 (reference)	
	High school/post high school	0.99 (0.69, 1.43)	0.95 (0.66, 1.37)	0.98 (0.83, 1.14)	1.00 (0.85, 1.18)
	Tertiary	1.07 (0.72, 1.60)	1.02 (0.70, 1.49)	0.94 (0.79, 1.11)	1.11 (0.93, 1.32)
Personal income	Lowest quartile	1 (reference)		1 (reference)	
	Medium-lowest quartile	1.18 (0.88, 1.59)	1.27 (0.96, 1.67)	1.06 (0.93, 1.22)	1.09 (0.94, 1.27)
	Medium-highest quartile	1.11 (0.80, 1.53)	1.20 (0.88, 1.63)	1.07 (0.91, 1.24)	1.08 (0.92, 1.27)
	Highest quartile	1.10 (0.75, 1.60)	1.31 (0.93, 1.85)	0.95 (0.81, 1.13)	1.18 (1.00, 1.40)^*^
Cannabis use status	Never user	1 (reference)		1 (reference)	
	Past user	0.85 (0.62, 1.16)	2.78 (2.07, 3.73)^**^	1.87 (1.68, 2.08)^**^	4.16 (3.75, 4.63)^**^
	Recent user	1.29 (0.46, 3.59)	14.13 (5.37, 37.20)^**^	3.19 (2.20, 4.61)^**^	33.74 (24.22, 47.01)^**^
Alcohol use status	Non-drinker/Low-risk drinker	1 (reference)		1 (reference)	
	High-risk drinker	1.40 (1.11, 1.77)^*^	2.12 (1.70, 2.65)^**^	1.27 (1.16, 1.39)^**^	1.57 (1.43, 1.72)^**^
Tobacco use status	Never smoker	1 (reference)		1 (reference)	
	Current smoker	0.96 (0.69, 1.34)	1.15 (0.84, 1.57)	1.44 (1.25, 1.66)^**^	1.47 (1.27, 1.70)^**^
	Ex-smoker	1.00 (0.78, 1.29)	1.26 (0.99, 1.59)	1.09 (0.98, 1.20)	1.12 (1.01, 1.24)^*^
Psychological distress^$^	Low level	1 (reference)		1 (reference)	
	Moderate level	0.88 (0.62, 1.25)	1.03 (0.74, 1.44)	1.11 (0.95, 1.30)	1.59 (1.36, 1.85)^**^
	High or very high level	0.83 (0.65, 1.05)	0.95 (0.76, 1.19)	1.10 (0.98, 1.22)	1.28 (1.14, 1.43)^**^
Cancer^%^	No	1 (reference)		1 (reference)	
	Yes	0.84 (0.53, 1.31)	0.99 (0.64, 1.52)	1.02 (0.83, 1.24)	1.06 (0.85, 1.33)
Chronicpain^§^	No	1 (reference)		1 (reference)	
	Yes	1.02 (0.72, 1.44)	1.44 (1.04, 2.00)^*^	0.96 (0.83, 1.10)	1.14 (0.98, 1.32)

*Odds ratios and 95% CIs are rounded to two decimal places*.

** *P-values < 0.001*;

* *P-values < 0.05*.

$ *Personal experience of psychological distress in the past month, categorized by Kessler Psychological Distress Scale (K10)*.

% *Being diagnosed or treated for cancer in the past 12 months*.

§ *Self-reported chronic pain in the past 12 months*.

## Discussion

The majority of Australian adults supported the decision to approve the use of cannabis for medicinal purposes. This high level of support is consistent with surveys from other countries that have implemented medical cannabis policies, with percentages of support at 91% in the USA and 78% in Israel (16). The high level of support agrees with a survey that found supporters generally believe the benefits of medical cannabis outweigh the potential side effects and so patients should have access to it (4). By contrast, only a third of Australians supported legalizing recreational cannabis. This supports the hypothesis that the public distinguishes between “medical cannabis” and “recreational cannabis” use, which affects public perceptions of the risks associated with these different reasons for uses and affects support for these different policies (5).

Females and persons over the age of 50 were more likely to support medical cannabis legalization, whereas, those who supported recreational cannabis use were more likely to be under the age of 30. The characteristics of Australian recreational cannabis supporters are similar to the supporters in other population, who are pre-dominantly younger (17). The different group of supporters for medical and recreational cannabis legalization perhaps partially reflect self-interest. Self-reported chronic pain was the strongest health factor associated with support for medical cannabis legalization in this study. Chronic pain was a common reason for medical use of cannabis as in previous studies (8, 9, 11). The sex and age correlates of support could reflect the fact that the prevalence of chronic pain is higher in females than males (18, 19) and increases with age. In contrast, persons suffering from moderate to very high level of psychological distress in the past month were more likely to support recreational cannabis. Although it is unclear whether the supporters would actually use cannabis if it became legal, using cannabis to cope with negative emotions is associated with elevated distress and cannabis use disorders (20). Therefore, assessment of cannabis related attitudes and motivation may be clinically important.

Personal experience with alcohol, tobacco and cannabis use were associated with supportive attitudes toward cannabis legalization and the association was especially strong with experience of cannabis use. Persons with recent cannabis experience were overwhelmingly more supportive of cannabis legalization than past users. Experience with cannabis may determine how a person perceives or interpret the benefits and risks associated with its use. The strong associations between recent cannabis use and support for legalization may have been driven by the reduced perception of risk and self-interest (21). Cannabis users would prefer cannabis use to no longer be a crime and to have easier access at lower prices. People who use cannabis by choice may also view the new medical cannabis policy as a validation for their beliefs about its benefits. Tobacco and excessive use of alcohol are widely recognized as harmful, with substantial public health and scientific efforts to reduce consumption and public harms over the years. The increased perception of medical cannabis as low in harm or beneficial may increase cannabis use. The epidemiology of cannabis use among cannabis users pre- and post-medical cannabis legalization warrants special attention.

There are several limitations in this study. As a cross-sectional survey, the study could only report associations. Data about history and frequencies of substance use were based on self-reports. Given the sensitive nature of these questions, there is a potential for social desirability bias despite the assurance of confidentiality given to survey participants. Also, views on legalization are likely to be shaped by a number of intersecting factors, such as views on criminal justice, personal liberty, and other aspects outside the scope of the survey, which should be considered when interpreting the results. Despite these weaknesses, this study provides an empirical examination of a wide range of factors that have shaped public opinion toward medical and recreational cannabis legalization in Australia.

In conclusion, the majority of Australians welcome the decision to legalize medical cannabis but many are cautious about legalizing recreational cannabis use. The different sociodemographic and clinical profile of supporters for medical and recreational cannabis policies suggests a potential interaction of self-interests and beliefs about cannabis. Perceptions of cannabis may be influenced by the subjective experience of cannabis or other substance use.

Future studies with data across different years is needed to verify the significance of these determinants consider the potential influence of age, period and cohort on the shifting attitude, and its association with the prevalence of cannabis use. The mechanism underlying the relationships between cannabis-related attitudes and cannabis legalization, and their links to the subjective intentions and decisions to use cannabis are not yet clear. Given that people are more inclined to support policies that work in favor of their personal interests, community-based surveillance of cannabis use may be needed as the liberalization of cannabis regulations increase access to and the availability of medicinal cannabis.

## Data Availability Statement

The data analyzed in this study is subject to the following licenses/restrictions: Permission to access the data is required by the data custodian. Requests to access these datasets should be directed to https://ada.edu.au.

## Ethics Statement

Ethical review and approval was not required for the study on human participants in accordance with the local legislation and institutional requirements. Written informed consent for participation was not required for this study in accordance with the national legislation and the institutional requirements.

## Author Contributions

VC, WH, and JL conceptualized the study. VC, GC, WH, CL, and JL contributed to the methodology. VC compiled the data, conducted the analysis, and completed the original draft. All other authors contributed to reviewing and editing the draft.

## Conflict of Interest

The authors declare that the research was conducted in the absence of any commercial or financial relationships that could be construed as a potential conflict of interest.
