# Supplementary material for: Personal Correlates of Support for Medical and Recreational Cannabis Legalization in Australia
Source: Front Psychiatry. 2021 Feb 25;12:551661. doi: 10.3389/fpsyt.2021.551661 (PMC7947688; doi:10.3389/fpsyt.2021.551661)
Supplement: Supplementary file 1 [file Table_1.docx]

**Appendix A: A comparison between AUDIT-C and 2016 NDSHS questions, and the scoring systems used for measuring risk levels of alcohol use status.**

| **AUDIT-C questions** | **Questions in 2016 NDSHS questionnaire** |
| --- | --- |
| How often do you have a drink containing alcohol?   \| Responses \| Scores \| \| --- \| --- \| \| Never \| 0 \| \| Monthly or less \| 1 \| \| 2-4 times / month \| 2 \| \| 2-3 times / week \| 3 \| \| 4+ times / week \| 4 \| | In the last 12 months, how often did you have an alcoholic drink of any kind?   \| Responses \| Scores \| \| --- \| --- \| \| Every day Less often \| 4 \| \| 5 to 6 days a week \| 4 \| \| 3 to 4 days a week \| 3.5 \| \| 1 to 2 days a week \| 2.5 \| \| 2 to 3 days a month \| 2 \| \| About 1 day a month \| 1 \| \| Less often \| 1 \| \| No longer drink \| 0 \| |
| How many standard drinks containing alcohol do you have on a typical day?   \| Responses \| Scores \| \| --- \| --- \| \| 1-2 \| 0 \| \| 3-4 \| 1 \| \| 5-6 \| 2 \| \| 7-9 \| 3 \| \| 10+ \| 4 \| | On a day that you have an alcoholic drink, how many standard drinks do you usually have?   \| Responses \| Scores \| \| --- \| --- \| \| 20 or more drinks \| 4 \| \| 16 – 19 drinks \| 4 \| \| 13 – 15 drinks \| 4 \| \| 11 – 12 drinks \| 4 \| \| 9 – 10 drinks \| 3.5 \| \| 7 – 8 drinks \| 3 \| \| 5 – 6 drinks \| 2 \| \| 3 – 4 drinks \| 1 \| \| 2 drinks \| 0 \| \| 1 drink \| 0 \| \| Half a drink \| 0 \| |
| How often do you have six or more drinks on one occasion?   \| Responses \| Scores \| \| --- \| --- \| \| Never \| 0 \| \| Less than monthly \| 1 \| \| Monthly \| 2 \| \| Weekly \| 3 \| \| Daily or almost daily \| 4 \| | Please record how often in the last 12 months you have had each of the following number of standard drinks in a day?   \| Responses/ Scores \| Everyday \| 5-6 days /week \| 3-4 days /week \| 1-2 days /week \| 2-3 days /month \| About 1 day /month \| Less often \| Never \| \| --- \| --- \| --- \| --- \| --- \| --- \| --- \| --- \| --- \| \| 20 or more standard drinks/day \| 4 \| 4 \| 3 \| 3 \| 2 \| 2 \| 1 \| 1 \| \| 11 – 19 standard drinks/day \| 4 \| 4 \| 3 \| 3 \| 2 \| 2 \| 1 \| 1 \| \| 7 – 10 standard drinks/day \| 4 \| 4 \| 3 \| 3 \| 2 \| 2 \| 1 \| 1 \| \| 5 – 6 standard drinks/day \| 4 \| 4 \| 3 \| 3 \| 2 \| 2 \| 1 \| 1 \| \| 3 – 4 standard drinks/day \| N/A \| N/A \| N/A \| N/A \| N/A \| N/A \| N/A \| N/A \| \| 1 – 2 standard drinks/day \| N/A \| N/A \| N/A \| N/A \| N/A \| N/A \| N/A \| N/A \| \| Less than 1 standard drink/day \| N/A \| N/A \| N/A \| N/A \| N/A \| N/A \| N/A \| N/A \| \| None \| N/A \| N/A \| N/A \| N/A \| N/A \| N/A \| N/A \| N/A \| |

**Appendix B. Description of the study population.**

| **Characteristics** | | **Overall sample for MML analysis  (N, weighted %)** | **Overall sample for RML analysis  (N, weighted %)** |
| --- | --- | --- | --- |
| Total | | 21,582 (100%) | 21,607 (100%) |
| Sex | Males | 9,798 (49.3) | 9,799 (49.3) |
|  | Females | 11,784 (50.7) | 11,808 (50.7) |
|  | Missing | 0 |  |
|  |  |  |  |
| Age group | 18-29 years old | 3,026 (21.7) | 3,027 (21.7) |
|  | 30-49 years old | 7,238 (35.5) | 7,249 (35.5) |
|  | 50+ years old | 11,318 (42.8) | 11,331 (42.8) |
|  | Missing | 0 |  |
|  |  |  |  |
| Marital status | Never married | 4,277 (24.2) | 4,269 (24.1) |
|  | Divorced / widowed / separated | 4,097 (12.1) | 4,097 (12.1) |
|  | Married | 13,075 (63.7) | 13,063 (63.8) |
|  | Missing | 133 | 178 |
|  |  |  |  |
| Employment status | Not in labour force | 8,720 (36.2) | 8,716 (36.2) |
|  | Unemployed / looking for work | 1,034 (5.9) | 1,033 (5.9) |
|  | Currently employed | 11,530 (57.9) | 11,513 (57.9) |
|  | Missing | 298 | 345 |
|  |  |  |  |
| Education attainment | Below high school | 1,623 (10.8) | 1,625 (10.8) |
|  | High school / post high school | 6,129 (43.9) | 6,125 (44.0) |
|  | Tertiary | 6,291 (45.2) | 6,282 (45.2) |
|  | Missing | 7,539 | 7,575 |
|  |  |  |  |
| Personal income | Lowest quartile | 4,480 (27.0) | 4,476 (27.0) |
|  | Medium-lowest quartile | 3,916 (21.6) | 3,909 (21.5) |
|  | Medium-highest quartile | 4,514 (26.6) | 4,508 (26.7) |
|  | Highest quartile | 4,228 (24.8) | 4,226 (24.8) |
|  | Missing | 4,444 | 4,488 |
|  |  |  |  |
| Cannabis use status | Never user | 13,692 (63.3) | 13,656 (63.3) |
|  | Past user | 5,810 (26.2) | 5,818 (26.2) |
|  | Recent user | 2,047 (10.6) | 2,046 (10.5) |
|  |  | 96 | 87 |
|  |  |  |  |
| Alcohol use status | Non-drinker / Low-risk drinker | 11,841 (55.1) | 11,847 (55.1) |
|  | High-risk drinker | 9,523 (44.9) | 9,543 (44.9) |
|  | Missing | 218 | 217 |
|  |  |  |  |
| Tobacco use status | Current smoker | 3,319 (15.4) | 3,325 (15.3) |
|  | Ex-smoker | 5,988 (24.6) | 5,999 (24.6) |
|  | Never smoker | 12,270 (60.1) | 12,278 (60.1) |
|  | Missing | 5 | 5 |
|  |  |  |  |
| Psychological distress | Low level | 14,745 (67.8) | 14,759 (67.8) |
|  | Moderate level | 4,309 (20.7) | 4,321 (20.7) |
|  | High or very high level | 2,415 (11.6) | 2,411 (11.5) |
|  | Missing | 113 | 116 |
|  |  |  |  |
| Cancer | Yes | 829 (3.8) | 828 (3.8) |
|  | No | 16,931 (96.2) | 16,943 (96.2) |
|  | Missing | 3,822 | 3,836 |
|  |  |  |  |
| Chronic pain | Yes | 2,540 (10.7) | 2,538 (10.7) |
|  | No | 17,792 (89.3) | 17,815 (89.3) |
|  | Missing | 1,250 | 1,254 |
